# Scale Characteristics of Intercultural Competence Measures and the Effects of Intercultural Competence on Prejudice

**DOI:** 10.3389/fpsyg.2021.686597

**Published:** 2021-07-19

**Authors:** Petia Genkova, Christoph Daniel Schaefer, Henrik Schreiber, Martina Rašticová, Jozsef Poor, Klara Valentinyi Veresné, Csilla Suhajda, Andrea Viszetenvelt, Jovana Bjekic

**Affiliations:** ^1^Department of Social and Economic Sciences, Osnabrück University of Applied Sciences, Osnabrück, Germany; ^2^Department of Law and Social Sciences, Mendel University in Brno, Brno, Czechia; ^3^Social Sciences Management and HR, Research Center, Hungarian University of Agriculture and Life Sciences, Gödöllö, Hungary; ^4^Institute for Medical Research, University of Belgrade, Belgrade, Serbia

**Keywords:** intercultural competence, cultural intelligence, multicultural personality, prejudice, intergroup relations, cooperation, cultural differences, measurement invariance

## Abstract

Due to proceeding globalization processes, involving a rise in mobility and international interdependencies, the frequency and relevance of intercultural contact situations increases. Consequently, the ability to deal effectively with intercultural situations is gaining in importance. However, the majority of studies on measures of intercultural competence focuses on Western Europe and the United States or cultures of the Far East. For the present study, previously understudied Eastern European (former communist) cultures were included, by sampling in Hungary, Serbia, and the Czech Republic, in addition to (the Central or Western European country) Germany. Thus, this study enabled comparisons of scale characteristics of the cultural intelligence scale (CQS), the multicultural personality questionnaire (MPQ), as well as the blatant and subtle prejudice scales, across samples from different cultures. It was also examined how the CQS and MPQ dimensions are associated with prejudice. To analyse scale characteristics, the factor structures and measurement invariances of the used instruments were analyzed. There were violations of configural measurement invariance observed for all of these scales, indicating that the comparability across samples is limited. Therefore, each of the samples was analyzed separately when examining how the CQS and MPQ dimensions are related to prejudice. It was revealed that, in particular, the motivational aspect of the CQS was statistically predicting lower prejudice. Less consistently, the MPQ dimensions of open-mindedness and flexibility were statistically predicting lower prejudice in some of the analyses. However, the violations of measurement invariance indicate differences in the constructs' meanings across the samples from different cultures. It is consequently argued that cross-cultural equivalence should not be taken for granted when comparing Eastern and Western European cultures.

## Introduction

The advance of globalization processes entails that various cultures are coming into contact with one another more frequently and more closely. Growing global interdependencies, whose challenging sides comprise, e.g., economic discrepancies and ecological crises, require international cooperation. Additionally, increased global mobility entails that many Western societies become more multicultural. With the increase in intercultural contact situations, intercultural competence gains in importance. In order to create and maintain inclusive European societies and international cooperation, commonalities and differences between European cultures need to be considered when investigating intercultural competences. However, insights in intercultural competence are, to a large degree, based on studies conducted in Western cultures and the Far East. The study underlying the present article was part of a larger research project, which focused on Eastern European countries and Germany, thus allowing cross-cultural comparisons of intercultural competence measures (Genkova, 2021). In the present article, the scale characteristics of the cultural intelligence scale (CQS), the multicultural personality questionnaire (MPQ), as well as the blatant and subtle prejudice scales were examined with samples from Hungary, Czech Republic, Serbia (Eastern European cultures) and Germany (a Western or Central European culture). The factor structure of these measures and the measures' invariances across these cultures were analyzed in order to evaluate the cross-cultural robustness of these widespread instruments for samples from Eastern European cultures. Additionally, we investigated which components of intercultural competence are associated with low prejudice, regarding low prejudice as a prerequisite for intercultural cooperation, to address economic, ecologic, and social challenges.

In the following sections of the theoretical introduction, at first the concept of intercultural competence is introduced. Then, previous results on its relationship to personality characteristics are sketched, before the conceptual and possible empirical relationships between intercultural competence and prejudice are addressed. The theoretical introduction is concluded by outlining the study which constitutes the basis for this article.

## Intercultural Competence

A common definition of intercultural competence has been popularized by Deardorff (2006), which specified as the aim of intercultural competence “behaving and communicating effectively and appropriately” in intercultural situations (Deardorff, 2004; in Deardorff, 2006). An established and widely used scale for the measurement of such an ability is the cultural intelligence scale (Van Dyne et al., 2012). Cultural intelligence is defined as the ability to function effectively in culturally diverse settings (Ang and Van Dyne, 2008). Being inspired by the intelligence model of Sternberg and Detterman (1986), the construct of cultural intelligence is conceptualized as being constituted by four components, namely metacognitive, cognitive, motivational, and behavioral (Ang et al., 2007; Ang and Van Dyne, 2008). Thus, the concept of cultural intelligence focuses on aspects which directly relate to intercultural interactions, categorized into the classical psychological dimensions of thinking, feeling, and acting. The metacognitive component of the cultural intelligence model refers to processes of acquiring knowledge about cultures. This includes the awareness of one's beliefs about cultural values and the adjustment of these beliefs during and after intercultural interactions. The cognitive component refers to knowledge of the values and practices of other cultures. The motivational component refers to devoting energy toward acquiring knowledge about other cultures and toward functioning in intercultural interactions. The behavioral component refers to possessing the behavioral capacities to act appropriately in intercultural interactions.

Despite the relative popularity of the construct of cultural intelligence, there is disagreement on the interrelationship of its components and accordingly on how to model them (Ang et al., 2007). The four components are in some models combined into a single factor; in other models the components feature as four correlated factors, while other authors represent the components by a bifactor model which contains both a general and a specific factor (see for an overview Rockstuhl and Van Dyne, 2018).

The CQS was initially validated with samples from both Singapore and the United States, including expatriates working in Singapore (Ang et al., 2007). In addition to Singaporean and US samples, subsequent evaluations covered, for example, Pilipino expatriates in Taiwan (Chen et al., 2011), expatriates of Taiwan companies (Lee and Sukoco, 2010), Korean students (Moon, 2010), and students of a Swiss military academy (Rockstuhl et al., 2011). Thus, the CQS was mainly employed in Western contexts and the Far East.

### Intercultural Competence and Personality Characteristics

While the cultural intelligence model targets aspects which directly relate to the thinking, feeling and acting in intercultural situations, other approaches focus on more distal correlates of appropriate intercultural behavior. A large family of studies examines which personality characteristics are related to intercultural competence. By a meta-analysis which investigated the relationships between personality characteristics and intercultural competence, Wilson et al. (2013) found small to medium effect sizes for all the traits from the Five Factor Model of personality, with neuroticism (negative correlation), openness/flexibility (positive correlation), and extraversion (positive correlation) having the strongest effects amongst these five. Further studies employed measures of adjustment and performances of expatriates, to analyse the effects of personality characteristics in a new culture. A meta-analysis revealed that extraversion, emotional stability (as the reverse of neuroticism), agreeableness, and conscientiousness, but not openness, are predictive of job performance abroad (Mol et al., 2005). Harari et al. (2018) point to a slightly different set of predictors of general adjustment, based on a meta-analysis with expatriates, namely extraversion, emotional stability, and open-mindedness. Thus, while the prediction of job performance and adjustment appear to demand a relatively specific range of constructs, all Big Five personality dimensions appear to be involved in more general operationalisations of cultural competence.

### The Concept of Multicultural Personality Characteristics

Based on the findings that personality dimensions affect the degree to which individuals are successful in intercultural situations, the Multicultural Personality Questionnaire (MPQ) was devised with the aim of measuring specifically personality characteristics that are particularly relevant for success in intercultural contact situations (Van der Zee and Van Oudenhoven, 2000, 2001; Van der Zee et al., 2013). The current version of this inventory aims at measuring five dimensions: cultural empathy, open-mindedness, emotional stability, social initiative, and flexibility. The theoretical background for the selection of these dimensions focuses on stress and challenges in intercultural situations. Van der Zee and Van Oudenhoven (2013) regard new intercultural contact situations as posing stress and challenges to individuals, while their five dimensions aim at measuring how successful individuals deal with these challenges.

Although personality characteristics might be modifiable only to a limited degree (in some conceptualisations by definition), there are indications that experiences and trainings can change cultural competence. It was suggested by a meta-analysis that studying abroad led to an increase in cultural awareness, operationalised as knowledge and reflections about cultural similarities and differences (Haas, 2018). Further support for the possible malleability of intercultural competence was revealed by the observation that individuals who studied abroad report increases in social abilities (Tracy-Ventura et al., 2016). Additionally, Morris and Robie (2001) found in their meta-analysis that cross-cultural training of expatriates had a positive effect on their performance and adjustment.

The MPQ was initially developed and validated in the Netherlands (Van der Zee and Van Oudenhoven, 2000). The scale was also used in Spain (Bobowik et al., 2011), in Belgium (Van der Zee et al., 2003), in France (Van Oudenhoven and van der Zee, 2002), in Italy (Leone et al., 2005), in the United States (Houtz et al., 2010), in Singapore (Leong, 2007), with Dutch and Frisian emigrants (Bakker et al., 2006), with employees of a Dutch multinational (Korzilius et al., 2011), with Western expatriates in Taiwan (Van Oudenhoven et al., 2003), or with Canadian expatriates (Simkhovych, 2009). In summary, most of the studies employed the MPQ with samples from Western cultures or the Far East.

### Intercultural Competence and Attitudes Toward Members of Outgroups

The conceptualization of these five dimensions implies overlaps with positive attitudes toward outgroups. In particular, open-mindedness is defined as an open and unprejudiced attitude toward different groups and cultural values (Van der Zee and Van Oudenhoven, 2001). Examining the relationship between open-mindedness and (low) prejudice can therefore be regarded as a question of criterion validity. Cultural empathy does not directly imply the absence of negative outgroup feelings, but comprises related concepts. It is defined as an interest in others and as the ability to understand and reflect the thinking, feeling and behaviors of cultural outgroup members (Van der Zee and Van Oudenhoven, 2001; Nesdale et al., 2012). This understanding and reflection is very similar to the concept of perspective taking, which, in turn, is empirically linked to lower prejudice (see Pettigrew and Tropp, 2008). Flexibility, defined as the ability to switch the own behavior and strategies in unfamiliar settings (Van der Zee and Van Oudenhoven, 2001; Nesdale et al., 2012), does not directly imply positive outgroup feelings either, but the switching of behavior to new settings is likely to be related to outgroup attitudes as well. If the ability of switching the own behavior is related to the willingness of adapting the behavior to other cultures, it should be associated with positive attitudes to other cultures. Empirically, it was revealed that the MPQ dimensions open-mindedness, flexibility, and cultural empathy are indeed negatively related to ethnic prejudice (Nesdale et al., 2012).

### From Intercultural Competence via Contact to the Reduction of Prejudice

In addition to these direct relationships between intercultural competence and prejudice, it is likely that intercultural competence is associated with more frequent and intense interactions with outgroup members. These interactions, in turn, are expected to be related to more positive intergroup attitudes. The MPQ was, similarly as the CQS, developed to measure or predict effectiveness in intercultural contact situations (Van der Zee and Van Oudenhoven, 2001; Van der Zee et al., 2013). To the extent that the measurement of the constructs possess validity, they should be related to rewarding experiences of intercultural contact. Studies on the CQS (Templer, Tay and Chandrasekar, 2006; Lee and Sukoco, 2010) and the MPQ (Van Oudenhoven et al., 2003) demonstrated that these measures are indeed related to cultural adjustment, while aspects cultural intelligence are also related to intercultural contact (Schwarzenthal et al., 2017). Intergroup contact, in turn, is associated with the reduction of prejudice, especially when contacts reduce anxiety and increase empathy (Pettigrew and Tropp, 2006, 2008). Intercultural competence could thereby not only reduce prejudice directly by the nature of its dimensions, but also indirectly via positive experiences in intercultural contact situations.

## Varieties of Prejudice

In the present article, relationships between intercultural competence dimensions and prejudice were examined. The operationalisation of prejudice drew on the seminal work of Meertens and Pettigrew (1997). These authors argue that societal norms have been rising in Northern America and Western Europe which prohibit the open expression of prejudice and discrimination (at least in some sections of these cultures). The entailed decline of open prejudice, however, does not necessarily imply that prejudice disappears, the authors argue, since prejudice can become expressed in more indirect or subtle ways. Meertens and Pettigrew (1997) employed seven samples from Western European countries to examine measures of blatant and subtle prejudice. They found that these measures for blatant and subtle prejudice evince correlations with each other between 0.48 and 0.70. When conducting confirmatory factor analyses, comparing a one factor model, a two factor models, and a second-order factor model, they found for three samples that a two factor model with uncorrelated measures fit best, while for four samples the second-order factor model had the best fit. Thus, the results suggest that the measures of blatant and subtle prejudice correlate with each other to varying degrees, depending on the context.

## The Present Study

In the present study, the scale characteristics of established measures of intercultural competence (i.e., the CQS and the MPQ) were analyzed in four national cultures. While previous studies focused in their employment of these instruments mostly on Western cultures and the Far East, the present study included cultures from Eastern Europe. In addition to Germany (as a Western or Central European culture), the Eastern European cultures of Hungary, the Czech Republic, and Serbia were included. These former communist countries might differ from Western countries in terms of their dealing with cultural diversity, as is reflected in their different approaches to immigration, which might be linked to their distinct histories.

The factor structures and invariances of the CQS and MPQ were compared across these four cultural contexts, thus contributing to the evaluation of these scales' generalizability. It was also examined how various aspects of these scales relate to prejudice. Thus, testing established scales and their intercorrelations in novel contexts enabled to compare both scale characteristics of intercultural competence and the relationship of intercultural competence to prejudice cross-culturally.

For the assessment of prejudice, measures of blatant and of subtle prejudice were employed, to ensure that both potential facets of prejudice are covered. We assumed that intercultural competence is negatively associated with prejudice. Specifically, it was hypothesized that open-mindedness, cultural empathy and flexibility are (each uniquely) negatively associated with prejudice. In line with the finding that flexibility and openness have unique predictive capacities (Wilson et al., 2013), flexibility and open-mindedness were operationalised separately. Additionally, it was hypothesized that there are indirect effects from multicultural personality dimensions and cultural intelligence components via contact quality on prejudice, since it was assumed that these dimensions would facilitate smooth intercultural interactions, which in turn should, in line with Pettigrew and Tropp (2006, 2008), reduce prejudice. The inclusion of samples from both Central European and Eastern European cultures enabled to evaluate to which degree measurement instruments which have been developed in Western cultures are robust in Eastern European cultures. These samples also allowed to compare cultures which are shaped by different traditions of immigration, potentially resulting in different attitudes toward foreigners.

In short, the hypotheses were the following:

Hypothesis 1: Components of cultural intelligence are (uniquely) negatively associated with prejudice.
Hypothesis 2: Open-mindedness, cultural empathy, and flexibility are (uniquely) negatively associated with prejudice.
Hypothesis 3: Dimensions of multicultural personality and intercultural competence have indirect effects via contact quality on prejudice.

## Method

### Respondents

To cover a broad range of cultures, allowing an intercultural comparison, samples in four cultural contexts of acceptable size could be recruited. There was no sufficient basis of previous results for assuming effect sizes needed for power analyses. A target number of 200 participants was reached in the following contexts: Czech Republic, Serbia, and Germany. Additionally, 194 participants were recruited in the Hungarian culture. In Germany, two samples were recruited, since data for two final theses were collected at the time of the project whose topics suggested the inclusion of the decisive measurement instruments of this project. Thus, there were five samples from four cultures altogether.

Sensitivity analyses were run using G^*^Power (Faul et al., 2007), to test the lower limit of effect sizes that could be detected in multiple regression analyses with 194 participants. In the multiple regression analyses specified for the present article (including the mediation analyses), we used seven to nine predictors. Sensitivity analyses for multiple regressions (fixed model) for single regression coefficients indicated that with 194 participants, seven predictors, and a significance level of 5% (one-tailed testing), a power of 95% would result from effect sizes of Cohen's *f*^2^ ≥ 0.056. For eight or nine predictors, effect sizes would also be ≥ 0.056. (Effect sizes *f*^2^ between 0.02 and 0.15 are termed small by Cohen, 1988).

Details for these five samples are presented in Table 1, listing the number of participants and socio-demographic information. All data analyses reported in this article are novel and have not been published elsewhere.

**Table 1:** Descriptive statistics for sociodemographic variables.

	**Number of participants**	**Age (M and SD)**	**Sex (proportion of female participants)**	**Education (proportion with college or university degree)**	**Country of birth (proportion of part. born in the country of the study)**	**Employment (proportion of participants in full-time employment)**
Hungary	194	30 years (SD = 8)	84%	91%	79%	62%
Czech Republic	235	21 years (SD = 2)	64%	87%	79%	7%
Serbia	209	22 years (SD = 12)	70%	31%	98%	Not available
Germany I	207	40 years (SD = 11)	75%	60%	87%	33%
Germany II	206	41 years (SD = 14)	69%	58%	90%	21%

### Procedure and Measures

In collaboration with partners in various European countries, nationwide convenience sampling was used, partially using an online version of the questionnaire and partially employing a paper-pencil version. For each sample, participants were informed about the anonymity and the voluntariness of the study prior to the start of the survey. They were informed that, for purely scientific purposes, the data could be processed anonymously. Participants were also informed that discontinuing the study would not result in any disadvantages for them. They were then asked for their informed consent.

In addition to sociodemographic information (e.g., age, gender, education), respondents provided ratings for the variables relevant to the analyses. All scales were translated to the main language of the respective country (i.e., Hungarian, Czech, Serbian, and German). For the translation, an expert committee approach was used. The items were translated from English into the respective language by a native speaker of the target language, then back-translated (following Brislin, 1990) by an English native speaker, evaluated by a committee for their fit, and if necessary, modified.

The scale characteristics of the used instruments were analyzed by evaluating measurement invariance, by calculating internal consistencies, and by conducting both confirmatory and exploratory factor analyses for the separate samples. To examine the effects of cultural competence on prejudice, multiple regression analyses with control variables were run, including mediation analyses.

The analyses for measurement invariance and the confirmatory factor analyses were specified in Mplus (Muthén and Muthén, 1998–2017), while exploratory factor analyses, internal consistency analyses, and multiple regressions were run in SPSS (IBM Corp. Released, 2019). For the confirmatory factor analysis, full-information maximum-likelihood was used for estimation.

#### Intercultural Competence

This construct was measured by the short form of the cultural intelligence scale (Van Dyne et al., 2008). This scale consists of four components, namely the meta-cognitive, the cognitive, the motivational, and the behavioral component. The metacognitive component has four items, the cognitive component has six items, while both the motivational component and the behavioral component have five items each. All of these items were used in the present study.

To analyse the statistical properties of this measure, confirmatory factor analyses were used, testing whether the four-component structure of the cultural intelligence scale could be replicated across the samples comprising the four cultures. We started with an assessment of configural measurement invariance across groups. That is, a confirmatory factor analysis of the cultural intelligence scale in a multiple group model was specified, stipulating the same factor structure across groups, while the factor loadings were allowed to vary between groups. We found that the model fit was below the bar of being satisfactory, so that the same factor structure should not be assumed across the examined groups [Comparative Fit Index [CFI] = 0.885; Tucker–Lewis Index [TLI] = 0.867; Root Mean Square Error of Approximation [RMSEA] = 0.092; Standardized Root Mean Residual [SRMR] = 0.067. For an acceptable fit, CFI and the TLI should be ≥0.9, while RMSEA and SRMR should be ≤0.08]. The five samples were then analyzed separately in confirmatory factor analyses. It was found that the fit was unsatisfactory in the Hungarian sample (CFI = 0.895; TLI = 0.879; RMSEA = 0.104; SRMR = 0.071), unsatisfactory in the Serbian sample (CFI = 0.828; TLI = 0.800; RMSEA = 0.120; SRMR = 0.069), satisfactory in the Czech sample (CFI = 0.922; TLI = 0.910; RMSEA = 0.066; SRMR = 0.067), and unsatisfactory, but close to the thresholds, in the German samples (CFI = 0.899; TLI = 0.883; RMSEA ≤ 0.079; SRMR ≤ 0.064). The fit indices for the confirmatory factor analysis are presented in Table 2, for all scale.

**Table 2:** Fit indices from the confirmatory factor analyses for each scale and sample.

	**Hungarian sample**	**Serbian sample**	**Czech sample**	**German sample I**	**German sample II**
**Cultural intelligence scale**					
CFI	0.895	0.828	0.922	0.899	0.899
TLI	0.879	0.800	0.910	0.883	0.883
RMSEA	0.104	0.120	0.066	0.079	0.078
SRMR	0.071	0.069	0.067	0.061	0.064
**Multicultural personality questionnaire**					
CFI	0.753	0.573	0.688	0.716	0.701
TLI	0.736	0.544	0.667	0.697	0.681
RMSEA	0.087	0.123	0.069	0.078	0.081
SRMR	0.114	0.128	0.083	0.099	0.097
**Contact quality**					
CFI	0.992	0.797	1.000	0.989	0.995
TLI	0.984	0.593	1.002	0.979	0.991
RMSEA	0.056	0.273	0.074	0.055	0.042
SRMR	0.018	0.086	0.006	0.032	0.021
**Prejudice: threat/rejection (reduced set of items)**					
CFI	0.780	0.925	0.929	0.962	0.967
TLI	0.559	0.740	0.858	0.924	0.933
RMSEA	0.189	0.186	0.132	0.138	0.143
SRMR	0.076	0.082	0.044	0.036	0.034
**Prejudice: blatant scale**					
CFI	0.843	Not available	0.907	0.923	0.959
TLI	0.793	Not available	0.878	0.899	0.946
RMSEA	0.102	Not available	0.084	0.100	0.084
SRMR	0.068	Not available	0.081	0.071	0.040
**Prejudice: subtle scale**					
CFI	0.906	Not available	0.875	0.982	0.924
TLI	0.868	Not available	0.824	0.975	0.894
RMSEA	0.098	Not available	0.094	0.037	0.097
SRMR	0.063	Not available	0.063	0.051	0.060

We repeated these analyses with the “MLR” as an estimator in Mplus, which uses maximum likelihood estimates with standard errors that are robust to violations of the normality assumption. The results remained largely the same, insofar as the unsatisfactory fits remained unsatisfactory, and the satisfactory fit remained satisfactory.

To explore the structure of this scale further, an exploratory principal component analysis was conducted. Applying the Kaiser-Guttman-Criterion (i.e., eigenvalues > 1), four components were extracted in all samples. When employing varimax rotation, these components corresponded (except for one item in the Czech sample) precisely to the four components identified by Van Dyne et al. (2008). The four factors explained ≥63.13% of the total variance in each sample. The internal consistencies of each of the four components were good for each of the five samples (Cronbach's α ≥ 0.808). The internal consistencies for all scales and samples can be found in Table 3.

**Table 3:** Internal consistencies for all scales and samples.

	**Hungarian sample**	**Serbian sample**	**Czech sample**	**German sample I**	**German sample II**
**Cultural intelligence scale's components**					
Metacognitive	0.925	0.831	0.883	0.840	0.820
Cognitive	0.911	0.808	0.874	0.858	0.820
Motivational	0.908	0.857	0.869	0.890	0.885
Behavioral	0.931	0.862	0.916	0.844	0.865
**Multicultural personality questionnaire**					
Open-mindedness	0.849	0.737	0.804	0.720	0.735
Cultural empathy	0.899	0.811	0.829	0.809	0.846
Flexibility	0.878	0.757	0.883	0.826	0.848
Orientation to action	0.829	0.758	0.800	0.843	0.806
Emotional stability	0.818	0.675	0.843	0.834	0.820
**Contact quality**					
(Contact quality)	0.811	0.950	0.733	0.792	0.830
**Prejudice: threat/rejection (reduced set of items)**					
(Prejudice, reduced set)	0.609	0.775	0.709	0.877	0.896
**Prejudice: blatant scale**					
(Blatant Scale)	0.640	Not available	0.752	0.860	0.908
**Prejudice: subtle scale**					
(Subtle scale)	0.750	Not available	0.724	0.796	0.862

We finally run a bifactor model, which was also suggested by some authors literature (Rockstuhl and Van Dyne, 2018). Bifactor models assume that each of the items are determined by both a general factor, which affects all items, and additionally by a sector-specific factor (in this case, the metacognitive, cognitive, behavioral, or motivational component). In other words, all items of the scale serve as indicators of (general) cultural intelligence, while the four components are additional latent variables which are competing influences on the items. In bifactor models, the various latent variables are not allowed to be correlated, so that they explain variance independently. We specified a bifactor model to test for configural invariance, but this model did not reach convergence. When running for each sample a separate analysis, all but one of the models reached convergence, the exception being the Serbian sample. The fits of the bifactor models (without the Serbian sample) were nearly acceptable (CFIs ≥ 0.925; TLIs ≥ 0.905; RMSEAs ≤ 0.090; SRMRs ≤ 0.081). These results suggest that the bifactor model has a better fit than the four-factor model, although not being satisfactory. When repeating the analyses with the MLR as an estimator, some fit indices in the Hungarian sample and the German samples deteriorated (Hungarian sample: CFI = 0.889, TLI = 0.872, RMSEA = 0.090, SRMR = 0.071; German samples CFIs ≥ 0.907; TLIs ≥ 0.892; RMSEAs ≤ 0.068; SRMRs ≤ 0064), while for the Czech sample they remained satisfactory. For this estimator, the Serbian sample converged and spawned an unsatisfactory fit (CFI = 0.806, TLI = 0.775, RMSEA = 0.117, SRMR = 0.069).

#### Multicultural Personality Questionnaire

From the MPQ, the short form with 40 items in total was employed. All of its dimensions and items were included (i.e., open-mindedness, cultural empathy, flexibility, orientation to action, and emotional stability; Van der Zee et al., 2013). Each of these five dimensions has eight items (All of these items were used in the present study).

Confirmatory factor analyses were run, to test whether the five-factor structure could be replicated in our samples. First, a test for configural invariance was conducted with all the items, specifying the model with five factors according to the assignment of the MPQ. The low fit of this model indicated a violation of configural measurement invariance (CFI = 0.676; TLI = 0.653; RMSEA = 0.090; SRMR = 0.105). Separate confirmatory factor analyses were specified for the samples. It was found that the fit was insufficient in the Hungarian sample (CFI = 0.753; TLI = 0.736; RMSEA = 0.087; SRMR = 0.114), insufficient in the Serbian sample (CFI = 0.573; TLI = 0.544; RMSEA = 0.123; SRMR = 0.128), insufficient in the Czech sample (CFI = 0.688; TLI = 0.667; RMSEA = 0.069; SRMR = 0.083), and insufficient in the German samples (CFI ≥ 0.701; TLI ≥ 0.681; RMSEA ≤ 0.081; SRMR ≤ 0.099). We repeated the analyses with the MLR as an estimator. The fit of all confirmatory factor analyses remained insufficient.

We then run principal component analyses, including all the MPQ (short form) items and separately for each of the samples, to openly examine the factor structure of the MPQ. For the Hungarian sample, this analysis spawned eight components; for the Serbian sample and both of the German samples, 10 components were extracted; for the Czech sample, 12 components resulted. This comparatively high number of factors as well as the variability suggests that a five factor solution is not appropriate for the samples studied.

For combining items to constructs, one option suggested by the results would have been to combine items idiosyncratically for each sample, guided by the principal component analyses. This procedure, however, would further reduce the comparability of results across samples and studies, as the meaning of constructs would change more substantially if they contained different sets of items.

After combining the items of open-mindedness to one scale, Cronbach's α was ≥0.675. For cultural empathy, Cronbach's α for the combined scale was ≥0.816; for flexibility, it was ≥0.757; for orientation to action, ≥0.734; and for emotional stability, ≥0.675.

#### Contact Quality

To measure contact with refugees, the General Intergroup Contact Quantity and Contact Quality Scale of Islam and Hewstone (1993) was used. We employed the five contact quality items of that scale.

Testing the one-factor model for configural invariance, the overall fit was close to acceptable but below the bar regarding the RMSEA (CFI = 0.954; TLI = 0.908; RMSEA = 0.138; SRMR = 0.047). The fit for the metric invariance model was insufficient (CFI = 0.894; TLI = 0.870; RMSEA = 0.163; SRMR = 0.132). To assess the fit of the one-factor solution for each sample, separate confirmatory factor analyses were run. The fit was good in the Hungarian sample (CFI = 0.992; TLI = 0.984; RMSEA = 0.056; SRMR = 0.018), insufficient in the Serbian sample (CFI = 0.797; TLI = 0.593; RMSEA = 0.273; SRMR = 0.086), very good in the Czech sample (CFI = 1.000; TLI = 1.002; RMSEA = 0.074; SRMR = 0.006), and good in the German samples (CFI ≥ 0.989; TLI ≥ 0.979; RMSEA ≤ 0.055; SRMR ≤ 0.032) (When being repeated with the MLR as an estimator, the analysis yielded analogous results).

In explanatory principal component analyses, one component was suggested (≥56.05% of variance explained) in all samples. Cronbach's α was ≥0.733.

#### Prejudice

The items for blatant and subtle prejudice of Pettigrew and Meertens (1995) were employed, to ensure coverage of various aspects of prejudice. The blatant prejudice scale consists of two sub-factors, namely threat/rejection, and intimacy. The threat/rejection factor is composed by six items; the intimacy scale comprises four items.

The subtle prejudice scale consists of the factors traditional values, cultural differences, and positive emotions. The traditional values and the cultural differences factors have four items each, while the positive emotions factor has two items (For the blatant prejudice scale, only five items were available in the case of the Serbian sample, which were the first five of the six items from the threat/rejection factor of Pettigrew and Meertens, 1995. The other items of the blatant and subtle prejudice scale were missing in the case of the Serbian sample).

The threat/rejection dimension was tested for configural measurement invariance across samples. Since the Serbian sample had a reduced set of items, we conducted a test for configural invariance based on this set of (five) items. The model testing for configural measurement invariance did not reach a satisfactory fit (CFI = 0.935; TLI = 0.870; RMSEA = 0.160; SRMR = 0.058). Separate confirmatory factor analyses were run for the samples. The fit was insufficient in the Hungarian sample (CFI = 0.780; TLI = 0.559; RMSEA = 0.189; SRMR = 0.076), insufficient in the Serbian sample (CFI = 0.925; TLI = 0.850; RMSEA = 0.186; SRMR = 0.082), insufficient in the Czech sample (CFI = 0.929; TLI = 0.858; RMSEA = 0.132; SRMR = 0.044), and satisfactory in the German samples, except for the RMSEA in one of the samples (CFI ≥ 0.962; TLI ≥ 0.924; RMSEA ≤ 0.143; SRMR ≤ 0.036). With the MLR estimator, all the analyses produced analogous results.

We additionally run tests for configural measurement invariance without the Serbian sample, which enabled to include all the variables. We tested the blatant and the subtle prejudice scales separately. For the blatant prejudice scale, the fit was (close to but) not satisfactory (CFI = 0.923; TLI = 0.898; RMSEA = 0.092; SRMR = 0.067). Separate confirmatory factor analyses were employed for the blatant scale, for each of the four samples. The fit was insufficient in the Hungarian sample (CFI = 0.843; TLI = 0.793; RMSEA = 0.102; SRMR = 0.068), insufficient in the Czech sample (CFI = 0.907; TLI = 0.878; RMSEA = 0.084; SRMR = 0.081), and at the thresholds of being acceptable in the German samples (CFI ≥ 0.923; TLI ≥ 0.899; RMSEA ≤0.10 0; SRMR ≤0.071). With the MLR estimator, all the analyses produced analogous results, except that the German samples reached satisfactory fits.

For the subtle prejudice scale, the configural invariance model caused estimation problems (resulting in non-positive definite latent variable covariance matrices). Separate confirmatory factor analyses were employed for the subtle scale and the four remaining samples. The fit was insufficient in the Hungarian sample (CFI = 0.906; TLI = 0.868; RMSEA = 0.098; SRMR = 0.063), insufficient in the Czech sample (CFI = 0.875; TLI = 0.824; RMSEA = 0.094; SRMR = 0.063), and at the thresholds of being acceptable in the German samples (CFI ≥ 0.924, TLI ≥ 0.894; RMSEA ≤ 0.097; SRMR ≤ 0.060). With the MLR estimator, all the analyses produced analogous results.

Analysing the structure of the blatant scale by exploratory principal component analysis resulted in four components for the Hungarian sample, while for the Czech and the German samples the analysis resulted in two components which (after Varimax rotation) by and large corresponded to the threat/rejection and intimacy factors of Pettigrew and Meertens (1995). Cronbach's α for the blatant scale was 0.709 for the Serbian sample (with the reduced set of items), and for the other samples with all items of the blatant scale ≥0.640. For the subtle prejudice scale, the principal component analysis resulted consistently in three components, which (after Varimax rotation) corresponded to the three factors of Pettigrew and Meertens (traditional values, cultural differences and positive emotions; 1995). Combined to one scale for subtle prejudice, Cronbach's α was ≥0.724 for the four samples.

## Results

Means, standard deviations, and correlations of the main variables are presented in Tables 4A–4E (one table for each sample). Since the preceding analyses on the factor structure of the constructs indicated violations of measurement invariance, each of the samples was analyzed separately, instead of running multiple group analyses. The analyses were furthermore run with manifest variables, to ensure equal weighing of each item across the various samples (We also had considered to change the used items in accordance with the exploratory principal component analyses, but this would have further reduced the comparability of the constructs with previous studies and across samples).

**Table 4A:** Hungarian sample: means, standard deviations, and intercorrelations for the main variables.

**Measure**	***M***	***SD***	**2**	**3**	**4**	**5**	**6**	**7**	**8**	**9**	**10**
1. Blatant prejudice	3.45	0.80	0.51^**^	−0.26^**^	−0.08	−0.08	−0.08	−0.01	−0.05	−0.12	−0.12
2. Subtle prejudice	4.16	0.78		−0.26^**^	0.09	−0.14	0.03	0.06	0.05	−0.06	−0.05
3. Contact quality	2.10	1.22			0.17^*^	0.14	−0.01	0.04	−0.01	0.17^*^	0.15^*^
4. Open-mindedness	3.73	0.71				−0.23^**^	0.61^**^	0.42^**^	0.29^**^	0.50^**^	0.40^**^
5. Flexibility	2.28	0.76					−0.31^**^	−0.17^*^	0.02	−0.05	−0.08
6. Cultural empathy	4.31	0.63						0.32^**^	0.22^**^	0.27^**^	0.23^**^
7. Meta-cognitive CQ	5.07	1.49							0.53^**^	0.52^**^	0.46^**^
8. Cognitive CQ	3.71	1.26								0.52^**^	0.50^**^
9. Motivational CQ	4.51	1.42									0.53^**^
10. Behavioral CQ	3.98	1.59									

*Higher mean scores indicate a higher level of the construct in question. The scores for blatant and subtle prejudice could range from 1 to 6, scores for the ingroup identification, contact quality and the dimensions of cultural intelligence could range from 1 to 7, while open-mindedness, flexibility, and empathy could range from 1 to 5. Owing to missing values, ns varied between 178 and 191*.

* *p ≤ 0.05;*

** *p ≤ 0.001 (all two-tailed)*.

**Table 4B:** Czech sample: means, standard deviations, and intercorrelations for the main variables.

**Measure**	***M***	***SD***	**2**	**3**	**4**	**5**	**6**	**7**	**8**	**9**	**10**
1. Blatant prejudice	3.51	0.87	0.33^**^	−0.36^**^	−0.29^**^	−0.07	−0.15^*^	−0.17^**^	−0.23^**^	−0.43^**^	−0.01
2. Subtle prejudice	4.60	0.64		−0.49^**^	−0.14^*^	−0.19^**^	0.03	−0.17^**^	−0.22^**^	−0.31^**^	0.00
3. Contact quality	3.40	1.83			0.16	0.12	0.09	0.29^*^	0.36^**^	0.43^**^	0.15
4. Open-mindedness	3.17	0.68				−0.12	0.33^**^	0.38^**^	0.37^**^	0.42^**^	0.31^**^
5. Flexibility	2.78	0.66					−0.15^*^	−0.09	−0.02	−0.04	−0.15^*^
6. Cultural empathy	3.92	0.57						0.33^**^	0.18^**^	0.24^**^	0.26^**^
7. Meta-cognitive CQ	4.16	1.25							0.43^**^	0.52^**^	0.40^**^
8. Cognitive CQ	3.99	1.05								0.50^**^	0.33^**^
9. Motivational CQ	4.19	1.35									0.41^**^
10. Behavioral CQ	3.75	1.33									

*The same score ranges and symbols as in Table 4A apply. Owing to missing values, ns varied between 63 and 235*.

* *p ≤ 0.05;*

** *p ≤ 0.001*.

**Table 4C:** Serbian sample: means, standard deviations, and intercorrelations for the main variables.

**Measure**	***M***	***SD***	**2**	**3**	**4**	**5**	**6**	**7**	**8**	**9**	**10**
1. Blatant prejudice	3.15	1.21		−0.12	−0.22^**^	0.05	−0.11	−0.17^*^	−0.18^**^	−0.20^**^	−0.11
2. Subtle prejudice	n/a	n/a			n/a	n/a	n/a	n/a	n/a	n/a	n/a
3. Contact quality	3.02	0.97			0.09	0.05	0.07	0.31^**^	0.24^**^	0.30^**^	0.20^**^
4. Open-mindedness	3.78	0.64				−0.13	0.60^**^	0.33^**^	0.21^**^	0.31^**^	0.25^**^
5. Flexibility	2.54	0.88					−0.30^**^	−0.05	−0.05	−0.07	−0.13
6. Cultural empathy	4.26	0.58						0.18^*^	0.04	0.29^**^	0.25^**^
7. Meta-cognitive CQ	5.05	1.51							0.49^**^	0.32^**^	0.34^**^
8. Cognitive CQ	4.39	1.43								0.31^**^	0.36^**^
9. Motivational CQ	5.46	1.36									0.49^**^
10. Behavioral CQ	3.96	1.85									

*The same score ranges and symbols as in Table 4A apply. Owing to missing values, ns varied between 201 and 209*.

* *p ≤ 0.05;*

** *p ≤ 0.001*.

**Table 4D:** German sample I: means, standard deviations, and intercorrelations for the main variables.

**Measure**	***M***	***SD***	**2**	**3**	**4**	**5**	**6**	**7**	**8s**	**9**	**10**
1. Blatant prejudice	2.28	0.93	0.64^**^	−0.41^**^	0.01	0.02	−0.11	0.02	0.10	−0.23^**^	−0.11
2. Subtle prejudice	3.75	0.79		−0.45^**^	−0.15	0.04	−0.12	−0.07	0.03	−0.28^**^	−0.15
3. Contact quality	3.90	1.26			0.20^*^	0.00	0.22^**^	0.17^*^	0.16^*^	0.40^**^	0.17^*^
4. Open-mindedness	3.43	0.47				0.03	0.43^**^	0.46^**^	0.46^**^	0.42^**^	0.25^**^
5. Flexibility	2.46	0.63					−0.13	−0.03	0.03	0.15	−0.02
6. Cultural empathy	4.19	0.50						0.36^**^	0.27^**^	0.42^**^	0.37^**^
7. Meta-cognitive CQ	4.60	1.30							0.40^**^	0.54^**^	0.49^**^
8. Cognitive CQ	3.76	1.20								0.37^**^	0.34^**^
9. Motivational CQ	4.98	1.21									0.59^**^
10. Behavioral CQ	4.78	1.29									

*The same score ranges and symbols as in Table 4A apply. Owing to missing values, ns varied between 144 and 199*.

* *p ≤ 0.05;*

** *p ≤ 0.001*.

**Table 4E:** German sample II: means, standard deviations, and intercorrelations for the main variables.

**Measure**	***M***	***SD***	**2**	**3**	**4**	**5**	**6**	**7**	**8**	**9**	**10**
1. Blatant prejudice	2.27	1.03	0.68**	−0.60**	−0.19**	−0.32**	−0.29**	−0.32**	−0.17*	−0.61**	−0.25**
2. Subtle prejudice	3.64	0.91		−0.54**	−0.28**	−0.25**	−0.25**	−0.31**	−0.21**	−0.54**	−0.31**
3. Contact quality	4.33	1.34			0.29**	0.30**	0.26**	0.35**	0.23**	0.60**	0.34**
4. Open-mindedness	3.51	0.51				0.04	0.35**	0.30**	0.26**	0.45**	0.15*
5. Flexibility	2.58	0.67					−0.06	0.15*	0.18*	0.32**	0.13
6. Cultural empathy	4.18	0.58						0.30**	0.19**	0.37**	0.28**
7. Meta-cognitive CQ	4.78	1.19							0.34**	0.55**	0.35**
8. Cognitive CQ	3.92	1.06								0.42**	0.35**
9. Motivational CQ	5.08	1.24									0.46**
10. Behavioral CQ	4.77	1.31									

*The same score ranges and symbols as in Table 4A apply. Owing to missing values, ns varied between 201 and 205*.

To test the hypothesis that cultural intelligence components lead to less prejudice, regression analyses were specified with the four CQS components as the predictors. Additional regression analyses were run with MPQ dimensions open-mindedness, cultural empathy, and flexibility as the predictors. For the regression analysing these MPQ dimensions, also orientation to action and emotional stability were included as control variables. In all analyses, age, sex (0 = female, 1 = male), and education (0 = no university or college degree, 1 = university or college degree) were included as control variables. As criteria, the measures of blatant and subtle prejudice were used (Since for the Serbian sample only the first five items of the six threat/rejection items were available, the results reported for the Serbian sample in following refer to the combination of these items).

### Hypothesis 1: Cultural Intelligence and Prejudice

Most consistent effects across all regression analyses were found for the motivational component. For both criteria, the pattern of the results was similar. The motivational component had a significant negative effect on all of the available criteria in all the samples, except for the Hungarian sample. That is, in the Czech sample, in the Serbian sample and the German samples, there was each a significant effect on blatant prejudice, and in the Czech sample and the German samples also on subtle prejudice (*B*s ≤ −0.145, *SE*s ≤ 0.082, βs ≤ −0.162, *t*s ≤ −2.03, *p*s ≤ 0.044) (For the Serb sample, there was no measure for subtle prejudice available). In the Hungarian sample, there was a marginally significant negative effect on subtle prejudice (*B* = −0.099, *SE* = 0.052, β = −0.180, *t* = −1.91, *p* = 0.058). In addition to the effects of the motivational component, in the Czech sample the behavioral component had a significant positive effect on both criteria (*B*s ≥ 0.089, *SE*s ≤ 0.045, βs ≥ 0.185, *t*s ≥ 2.59, *p*s ≤ 0.010).

[Since the blatant and subtle prejudice scales evinced insufficient scale characteristics, as reported above, the sub-dimensions of these scales were additionally used as separate criteria (i.e., threat/rejection, intimacy, traditional values, cultural differences, and positive emotions). Across all five sub-dimensions, the motivational component had the most consistent effects. In the Czech, the Serbian, and the German samples, the motivational component had significant effects on all of the five sub-dimensions, while in the Hungarian sample it had a significant effect on intimacy, cultural differences, and positive emotions (*p*s ≤ 0.046)].

The two main regression analyses with blatant and subtle prejudice as criteria were repeated without the demographic variables as control variables to check for the robustness of the results. All main effects of the four components were replicated and no additional effect emerged, indicating that the main results were robust to the inclusion or exclusion of the demographic control variables.

### Hypothesis 2: Cultural Personality Characteristics and Prejudice

For the MPQ-predictors open-mindedness, cultural empathy, flexibility, as well as the for the control variables of orientation to action and emotional stability, some significant effects on the prejudice measures were found, but the pattern was less pervasive. Open-mindedness had a negative effect on the available criteria in the Czech sample and the Serbian sample, and it also had a significant negative effect on subtle prejudice in one of the German samples (*B*s ≤ −0.159, *SE*s ≤ 0.175, βs ≤ −0.170, *t*s ≤ −2.18, *p*s ≤ 0.030). Flexibility had a negative effect on both criteria in the Hungarian sample and in one of the German samples, and on subtle prejudice in the Czech sample (*B*s ≤ −0.197, *SE*s ≤ 0.063, βs ≤ −0.205, *t*s ≤ −2.65, *p*s ≤ 0.009). Cultural empathy had a negative effect on both criteria in a German sample (Bs ≤ −0.288, SEs ≤ 0.114, βs ≤ −0.185, ts ≤ −2.52, ps ≤ 0.013). Orientation to action had a positive effect on subtle prejudice in the Hungarian sample (Bs = 0.284, SEs = 0.080, βs = 0.282, ts = 2.56, ps < 0.001). There were no significant effects of emotional stability.

(It was also tested whether there were effects of the predictors on the sub-dimensions of the prejudice scales. The resulting pattern of effects was similar to the previous results. The two main regression analyses with blatant and subtle prejudice as criteria were also repeated without the demographic variables as control variables to check for the robustness of the results. The pattern of results remained largely the same).

### Hypothesis 3: Indirect Effects of Cultural Competence via Contact on Prejudice

Mediation analyses were conducted, testing whether there are indirect effects of the motivational component, open-mindedness, cultural empathy and flexibility via the perceived quality of contact with refugees on prejudice. We used the SPSS Macro Process (2016) from Hayes to compute indirect effects. Significant indirect effects were found for the motivational component via contact quality on both blatant and subtle prejudice in the Czech sample and both German samples (*b* ≤ −0.068, 95% CI [≤ −0.174, ≤ −0.001], bias-corrected bootstrapping with 10,000 resamples). For open-mindedness, there were indirect effects on both criteria in the Hungarian sample and in one of the German samples (*b* ≤ −0.068, 95% CI [≤ −0.116, ≤ −0.166], 10,000 resamples). The pattern was the same for flexibility, having indirect effects on both of the criteria in the Hungarian sample and one of the German samples (*b* ≤ −0.043, 95% CI [≤ −0.108, ≤ −0.007], 10,000 resamples). For cultural empathy, there were indirect effects on both criteria in both of the German samples (*b* ≤ −0.133, 95%-CI [≤ −0.270, ≤ −0.011], 10,000 resamples).

(Further questions on details of the dataset can be directed to the corresponding author of the article).

## Discussion

Established measurement instruments for assessing the statistical characteristics of these instruments and their interrelations were employed in various cultures, including Eastern European cultures. Thereby, instruments which were developed and evaluated largely in Western cultures were subjected to a cross-cultural test of robustness and consistency. Since there was no measurement invariance across cultures, the samples were investigated separately, instead of in multiple group analyses. As Eastern European cultures have a history different from Western European cultures, not least due to the period of communism, the present study could analyse and compare samples stemming from previously understudied cultural contexts. The study thus contributes to the evaluation of the cross-cultural reliabilities and factor structures of the examined scales.

### Cultural Intelligence Scale: Scale Characteristics and Correlates

This scale consists of four components: The metacognitive component, which covers the awareness of one's beliefs about cultural values and the adaptation of these beliefs. The cognitive component consists of the knowledge of other cultures. The motivational component includes devoting energy toward functioning in intercultural interactions. The behavioral component refers to possessing the behavioral capacities to act appropriately in these interactions.

When examining the cultural intelligence scale (CQS) for measurement invariance, configural measurement invariance was not found across the samples. Further confirmatory factor analyses which were run separately on the samples indicated that the stipulated factor structure of the CQS with four components (metacognitive, cognitive, motivational, and behavioral) was only found in the Czech sample, while in the other samples the fits were not satisfactory. The lowest fits were observed in the Hungarian and the Serbian samples. Thus, the analyses suggest that the meaning of the items or the underlying constructs were not equivalent across the samples. However, four components were consistently extracted across the samples in exploratory principal component analyses. These four components corresponded to the components for whose measurement the instrument had been designed. The extraction of four components suggests that it is highly questionable to combine the items of the cultural intelligence scale into one dimension.

#### The Possible Conceptualization of CQS as a Bifactor Model

The extraction of four components is particularly informative when viewed in the light of the partially low fits observed in the confirmatory factor analyses. The combination of these results suggests that a four factor structure appears to be appropriate, but that the residuals of some items are correlated. This pattern is consistent with a bifactor model as represented by Rockstuhl and Van Dyne (2018), in which both a general factor of cultural intelligence and four specific factors are represented. A bifactor model reached acceptable fits in (only) some of our analyses. The choice of model should, however, not only be based on the fit values, but also on theoretical considerations about the relationships between the components of the CQS. In a bifactor model, the components (or sector-specific factors) are specified as uncorrelated, so that all the commonalities between items across components must be explained by the general factor. Thus, a bifactor model of CQS is incompatible with the assumption of overlaps, for example, between the motivational and the behavioral component. Future research should reflect on the theoretical underpinnings of a bifactor model and should elucidate to what extent a bifactor model possesses better concurrent and predictive validity than the separate four components.

#### CQS and Prejudice

Due to the partially insufficient fits of the confirmatory factor analyses, the combination of the items to the CQS components is problematic. The respective items were average to ensure equal weighting of the items across the samples. Although this combination is suboptimal in the light of some of the confirmatory factor analyses, this procedure came closest to enable comparability across the samples and with previous results.

The motivational component had a consistent effect on prejudice across most samples (Thus, hypothesis 1, which stated that components of cultural intelligence are uniquely associated with prejudice, could only be corroborated for this component). The motivational component is associated with a reduction of blatant prejudice in all but one of the examined cultures. The motivational component also had an effect on subtle prejudice in two (out of four) possible samples. The observation that the motivational component was more predictive of the blatant prejudice scale (than of the subtle prejudice scale) suggests a conceptual similarity of the constructs, which points to the possibility that both scales could be influenced by social desirability tendencies. Alternatively, or additionally, the motivational component, which has at its conceptual core the inclination of devoting attention to (and being optimistic about) functioning in intercultural settings, could be an indication of low intergroup anxiety, which in turn should reduce prejudice (see Pettigrew and Tropp, 2006), whilst fear might be associated in particular with more extreme and blatant forms of prejudice.

Indirect effects were also examined. There was an indirect effect of the motivational component via contact quality on prejudice in the Czech and both German samples (Thus, hypothesis 3, stating that dimensions of multicultural personality and intercultural competence have indirect effects via contact quality on prejudice, could be corroborated for the motivational component). This suggests that (part of) the association between the motivational component and prejudice could be explained by different levels of experiences with intercultural contact situations in the respective contexts (e.g., Hungary vs. Germany). Future studies should explore this relationship further. Given that the study was cross-sectional, it needs yet to be established in which causal relationship these variables are positioned. It is, for example, conceivable that low prejudice leads to contacts and to a higher motivation to meet outgroups (instead of vice versa). There is also the likely possibility that attitudes, contact experiences, and motivation have mutual reinforcing relationships with one another. Future studies should longitudinally examine to what degree prejudice and contact quality are antecedents or consequences of the ability to function effectively across cultures, and should also explore facilitating conditions.

### Multicultural Personality Questionnaire: Scale Characteristics and Correlates

When examining the multicultural personality questionnaire (MPQ) for measurement invariance, configural measurement invariance was not found across the samples. The subsequent separate confirmatory factor analyses consistently yielded insufficient fits, indicating that the items do not form constructs in accordance with the MPQ model (open-mindedness, cultural empathy, flexibility, social initiative, emotional stability) in these samples.

#### MPQ and Prejudice

The items were still combined to the dimensions posited by the MPQ. It was found that open-mindedness and flexibility were the dimensions that most frequently predicted lower prejudice measures, although not consistently across samples (Thus, hypothesis 2, stating that open-mindedness, cultural empathy, and flexibility are uniquely associated with prejudice, could only be partially corroborated).

Since open-mindedness and flexibility had unique effects in these instances, a pertinent implication is that flexibility should be operationalised separately from openness, instead of being subsumed into one category. This conceptual separation is consistent with the findings of Wilson et al. (2013).

Cultural empathy was occasionally related to the reduction of prejudice. There were also indirect effects of open-mindedness, flexibility, and cultural empathy via contact quality on the reduction of prejudice in some of the samples, again suggesting that parts of the relationships could be explained by contact quality (Thus, hypothesis 3, stating that dimensions of multicultural personality and intercultural competence have indirect effects via contact quality on prejudice, could only be partially corroborated). Future studies should examine these relationships longitudinally. It could also be further explored to what degree open-mindedness, flexibility, and cultural empathy can be fostered by intercultural trainings.

### Scale Characteristics of the Used Instruments and Implications

The analyses on the scale characteristics of the MPQ and the prejudice scales shed doubt on the practice of combining the items into the scales in accordance with the structure proposed by the respective models. For the CQS, confirmatory factor analyses indicated fits that were satisfactory or nearly satisfactory for three of the studied samples. For the MPQ, however, fits of confirmatory factor analyses were insufficient for all samples. For the prejudice scales, fits were insufficient for three of the samples.

It is possible that the low fits in the confirmatory factor analyses of this study relate to specificities in the translation of the items or to methodological specificities. Since, however, the translation processes was double-checked by back-translation and involved native speakers of the respective target languages, it is unlikely that the denotation of the items differed widely from the originals across cultures. More likely is that specific connotations varied, potentially also involving that evaluative characteristics of the items differed across cultural contexts. The observation that confirmatory factor analyses spawned low fits particularly for Eastern European samples merits further examinations of scale characteristics in future studies. Particularly inventories which aim at cross-cultural validity should not only be tested in Western cultures and cultures of the Far East, but should also be evaluated in a broad spectrum of cultures, including Eastern European contexts. A broad variety of cultural contexts should be taken into account in scale development and when examining the potential for reducing prejudice and improving intercultural contacts.

Regarding the prejudice scales, also the number of components extracted in the exploratory main component analyses varied across the cultures. Remarkably, Meertens and Pettigrew (1997) themselves report that they found between one and three components across their samples in exploratory analyses for the blatant scale, which indicates some sample- or culture-specific variation. These findings on the number of sub-components put the combination of the sub-factors into a blatant and a subtle scale into question. It should therefore be considered to analyse the sub-factors separately.

### Possible Sources of the Observed Differences Across the Samples

There is the possibility that differences between the samples are partially attributable to differences in demographic variables. Age, in particular, could be related to different degrees of intercultural competence, as age is likely to be associated with intercultural experiences. In the regression analyses, age, gender, and education were included as control variables, so that spurious correlations between intercultural competence dimensions and prejudice, caused by these demographic variables, are unlikely. However, the strength of the association between intercultural competence and prejudice might be moderated by these demographic variables. Due to the theoretical importance of age (as being related to life experiences and cohort effects), additional regression analyses with product terms of age and intercultural competence dimensions were run, to check for potential moderations by age. A separate analysis for each product term and for each sample was conducted, with blatant and subtle prejudice as criteria. Negative interaction effects indicate here that the prejudice-reducing effects of intercultural competence dimensions are stronger for older participants than for younger participants, while positive interaction effects indicate that the effects are stronger for younger people. For one of the German samples, there were significant positive interaction effects of (age times) the cognitive and the motivational component of the CQS on blatant prejudice, and of the metacognitive, cognitive, and motivational component on subtle prejudice (*p* ≤ 0.048). For the Hungarian sample, there was a negative interaction effect of the cognitive component on subtle prejudice, and of flexibility on subtle prejudice (*p* ≤ 0.030). For the Czech sample, there was a positive interaction effect of open-mindedness and a negative effect of flexibility on subtle prejudice (*p* ≤ 0.045). Against the backdrop of the large number of moderation analyses, these interaction effects with both positive and negative signs suggest that there are no systematic moderation effects of age on the intercultural competence dimensions in any consistent direction. There remains, however, the possibility that life experiences (that are not sufficiently represented by age) played a role for the results in our samples. In more general terms, differences in demographic characteristics between samples imply the possibility that the strength of relationships between constructs is influenced by the respective composition of the samples.

Since the German samples differed in terms of some relationships between the intercultural competence dimensions and prejudice, some results appear to be of limited robustness. Differences in demographic factors might be partially responsible for these differences in some relationships.

Variations across the five samples that were observed for the blatant and subtle prejudice scales could not only stem from differences in demographic factors or experiences with foreigners, but also from the specific response-formats of these scales. Meertens and Pettigrew (1997) report a response format that reach from high to low (e.g., from “strongly agree” to “strongly disagree”). In the present study, the same response scheme was used. For the MPQ and the CQS, the original response scales were used as well, which reach from low to high. The unconventional response format of the prejudice scales might therefore have been confusing for some participants, possibly contributing to variations across cultures, depending on specific experiences with certain scale formats.

## Conclusion

Given that the MPQ and the prejudice measures are rather established scales which are frequently regarded as being applicable across cultures, the found lack of measurement invariances raises important questions that reach far beyond the present study. While we cannot rule out particularities in the studied samples or the methodological contexts, differences in the cultural contexts are prominent potential explanations for the observed deviations. While the similarity of the items' denotations across samples should have been ensured by the involvement of native speakers, possible differences in the meaning of scale items on the level of connotations might imply that evaluative characteristics of the items differed across cultural contexts. Additionally, or alternatively, differences between samples might be related to differences in regional experiences and social desirability between cultures. Differences, for example, in the proportion of immigrants and the resulting differences in the experience with intercultural contacts might foster variability in item intercorrelations, insofar as some of the item values might reflect differences in being intuition-vs. experience-based. Additionally, the political climate and status of ethno-cultural minorities in the national cultures are likely to influence social desirability, which could affect certain items of a scale more strongly than others.

The present study also points to the possibility that intercultural competence facilitates intercultural contacts, which might result in less prejudice and more cooperation. Future studies with more robust scales and longitudinal designs are needed for a corroboration of these effects. If these effects can be corroborated, they will imply that the fostering of intercultural competence in education and training can constitute an important building block for the reduction of prejudice in intercultural contact situations. This is of high importance in the context of increasing international interdependencies and the necessity of intercultural cooperation for jointly addressing international challenges. The global interdependencies involve both chances and risks for cooperation. Inherent in diversity is the danger of fragmentation along fault lines into small sub-groups with sharp borders, which can fuel mistrust and alienation. An increasing pluralisation thus implies that cultural differences in combination with misunderstandings and competition can lead to conflicts. “Us vs. them”-thinking and conflict escalation is a likely consequence when prejudice prevails.

When the necessity of cooperation on global challenges on health, the global economy, economic inequalities or the climate is accepted, skills are required that enable to deal with global differences in productive ways. The reduction of prejudice and mutual understanding are sine qua non for successful intercultural cooperation to address economic, ecologic, and social challenges. The present study suggests that the motivational component of cultural intelligence, contact, and (in some contexts) open-mindedness and flexibility are associated with low prejudice. Thus, the motivation for mutual understanding, intercultural competence, and intergroup contact could constitute important steps for the way to a more productive global cooperation.

## Data Availability Statement

The raw data supporting the conclusions of this article will be made available by the authors, without undue reservation.

## Ethics Statement

Ethical review and approval was not required for the study on human participants in accordance with the local legislation and institutional requirements. The patients/participants provided their written informed consent to participate in this study.

## Author Contributions

PG, MR, JP, KV, CS, AV, and JB contributed to conception and design of the study. PG organized the database. CS performed the statistical analysis and wrote the first draft of the manuscript. PG and HS contributed to the editing of the manuscript. All authors read and approved the submitted version.

## Conflict of Interest

The authors declare that the research was conducted in the absence of any commercial or financial relationships that could be construed as a potential conflict of interest.
